# Multi-site metabolomics reveals one-carbon and placental steroid disruption in maternal pre-pregnancy obesity linked to adverse pregnancy outcomes

**DOI:** 10.3389/fmed.2026.1904730

**Published:** 2026-10-01

**Authors:** Ke Ye, Yan Liu, Jinghui Zou, Hangyu Wu, Yisheng Zhang

**Affiliations:** Department of Obstetric, The Affiliated Lihuili Hospital of Ningbo University, Ningbo, China

**Keywords:** folate, maternal-fetal metabolic axis, metabolomics, one-carbon metabolism, pre-pregnancy obesity, steroid hormones, vitamin b12

## Abstract

**Purpose:**

This study characterized metabolomic profiles of maternal adipose tissue, placenta, and cord blood in pre-pregnancy obesity and explored links to adverse pregnancy outcomes (APOs).

**Methods:**

From a prospective cohort (Oct 2024–Oct 2025, *n* = 1,335), 25 obese (pre-pregnancy BMI ≥ 28 kg/m^2^) and 25 control women undergoing cesarean section were included. Visceral adipose, placenta, and umbilical vein plasma were analyzed using UHPLC-MS/MS. Metabolite differences were analyzed using Random Forest modeling, OPLS-DA, KEGG pathway enrichment analysis, and correlation analysis with clinical indicators, including gestational diabetes mellitus (GDM), hypertensive disorders of pregnancy (HDP), preterm birth, macrosomia, postpartum hemorrhage (PPH), and various laboratory parameters. In an independent external cohort, mRNA expression levels of key steroidogenic enzymes in placental tissues were verified by qRT-PCR.

**Results:**

The obesity group showed significantly higher incidences of GDM, HDP, PPH and higher neonatal weight. Vitamin B12 and folic acid were key discriminators across all compartments. Adipose tissue metabolites were enriched in arginine/proline, pentose phosphate, and cysteine/methionine metabolism. Placenta was enriched in steroid hormone biosynthesis pathways. Cord blood additionally showed enrichment in TCA cycle, glutamatergic and serotonergic synapses. Key compartment-specific metabolites included etiocholanolone (adipose), 16-glucuronide-estriol (placenta), and 5*α*-androstan-3-one (cord blood), all of which correlated with clinical indicators. The mRNA expression changes of key steroidogenic enzymes in placental tissues paralleled the alterations in their corresponding metabolite abundances.

**Conclusion:**

Pre-pregnancy obesity-related adverse pregnancy outcomes are driven by a disrupted maternal-fetal metabolic axis characterized by three core features: global dysfunction of one-carbon metabolism exemplified by vitamin B12 and folic acid abnormalities, placental steroid hormone biosynthesis abnormalities as the central hub, and disturbances in fetal energy and neurotransmitter metabolism as the downstream consequence. Monitoring these metabolites may enable early risk identification and guide targeted interventions for placental steroid metabolism.

## 1 Introduction

The global prevalence of obesity is increasing rapidly. According to the 2025 World Obesity Map, the proportion of overweight and obese adults worldwide is projected to rise from 35% to 50% by 2030 (1, 2). Obesity represents the metabolic disorder with the highest disease burden among women of reproductive age, consistently ranking first in age-standardized disability-adjusted life years (DALYs) (3). In China, data from 2024 indicates that the prevalence of obesity among childbearing period female was 6.75%, with an average annual growth rate of 4.63% (4). This trend signifies a continuous increase in the population experiencing pre-pregnancy obesity.

Pre-pregnancy obesity significantly increases the risk of adverse pregnancy outcomes (APOs), including gestational diabetes mellitus (GDM), hypertensive disorders of pregnancy (HDP), macrosomia, preterm birth, increased rates of cesarean delivery, and long-term offspring metabolic diseases (5–8). A prospective cohort study revealed a positive correlation between pre-pregnancy body mass index (BMI) and macrosomia; notably, among pregnant women with GDM, the effect of pre-pregnancy BMI on macrosomia was stronger, with each one-unit increase in BMI associated with a 24% increased risk (9). Additionally, increased adiponectin ubiquitination and decreased total adiponectin levels were observed in the adipose tissue of obese pregnant women, exacerbating maternal insulin resistance (10). Mother is the sole provider of lipids for the fetus, with fatty acids and/or metabolites derived from long-chain fatty acids being selectively transported across the placenta throughout gestation. Research indicates that visceral adipose tissue (VAT) thickening in obese pregnant women, coupled with excessively high or low levels of lipid metabolites such as phospholipids and steroids in maternal serum, may predispose to macrosomia (11, 12). These findings underscore the intricate regulation of fetal growth by maternal metabolic homeostasis. However, the specific metabolic mechanisms remain unclear, and existing research is largely confined to single tissues. Current evidence is insufficient evidence to demonstrate a systemic transfer network of metabolite changes between the mother, placenta, and fetus, leading to a lack of holistic understanding of the maternal-offspring metabolic axis.

To address this gap, this study prospectively enrolled a cohort of women with pre-pregnancy obesity and normal-weight pregnant women. Utilizing untargeted metabolomics, we simultaneously analyzed maternal adipose tissue, placental tissue, and umbilical cord blood serum to assess the characteristics of the maternal-fetal metabolic axis and its association with offspring birth weight. Our aim is to elucidate how obesity-induced metabolic dysfunction and chronic inflammation are transmitted from mother to fetus and to identify potential intervention points. This research provides new insights into the maternal-fetal transmission mechanisms underlying obesity-related macrosomia and offers a basis for optimizing perinatal management strategies for individuals with pre-pregnancy obesity.

## 2 Materials and methods

### 2.1 Study design and participant enrollment

This prospective cohort study initially included 1,335 pregnant women delivered at The Affiliated Lihuili Hospital of Ningbo University between October 1, 2024, and October 31, 2025. After applying exclusion criteria—removing individuals with multiple gestation(*N* = 2), assisted reproductive techniques (ART, *N* = 6), pregestational diabetes mellitus (PGDM, *N* = 28), hypertension (*N* = 17), vaginal delivery(*N* = 764) or those with incomplete clinical data(*N* = 339), a final analytical sample of 179 participants was retained.

And we obtained a clinical research cohorts based on whether their pre-pregnancy BMI reached 28 kg/m^2^, including 70 women in the obesity group (*N* = 70) and 109 women in the control group (*N* = 109). In addition, we selected 179 participants who met the following criteria: (1) cesarean section delivery with collection of adipose tissue, placental tissue, and umbilical cord blood during the procedure. (2) agree to metabolomics analysis. (3) acceptable sample quality [exclusions: insufficient sample size; visible hemolysis, coagulation, or lipemia; degradation (e.g., storage at room temperature for more than 4 h); labeling errors; contamination during collection or transportation]. A total of 50 participants were included in a metabolomic research cohorts, consisting of the obesity group (*N* = 25) and the control group (*N* = 25) (Figure 1).

**Figure 1:**
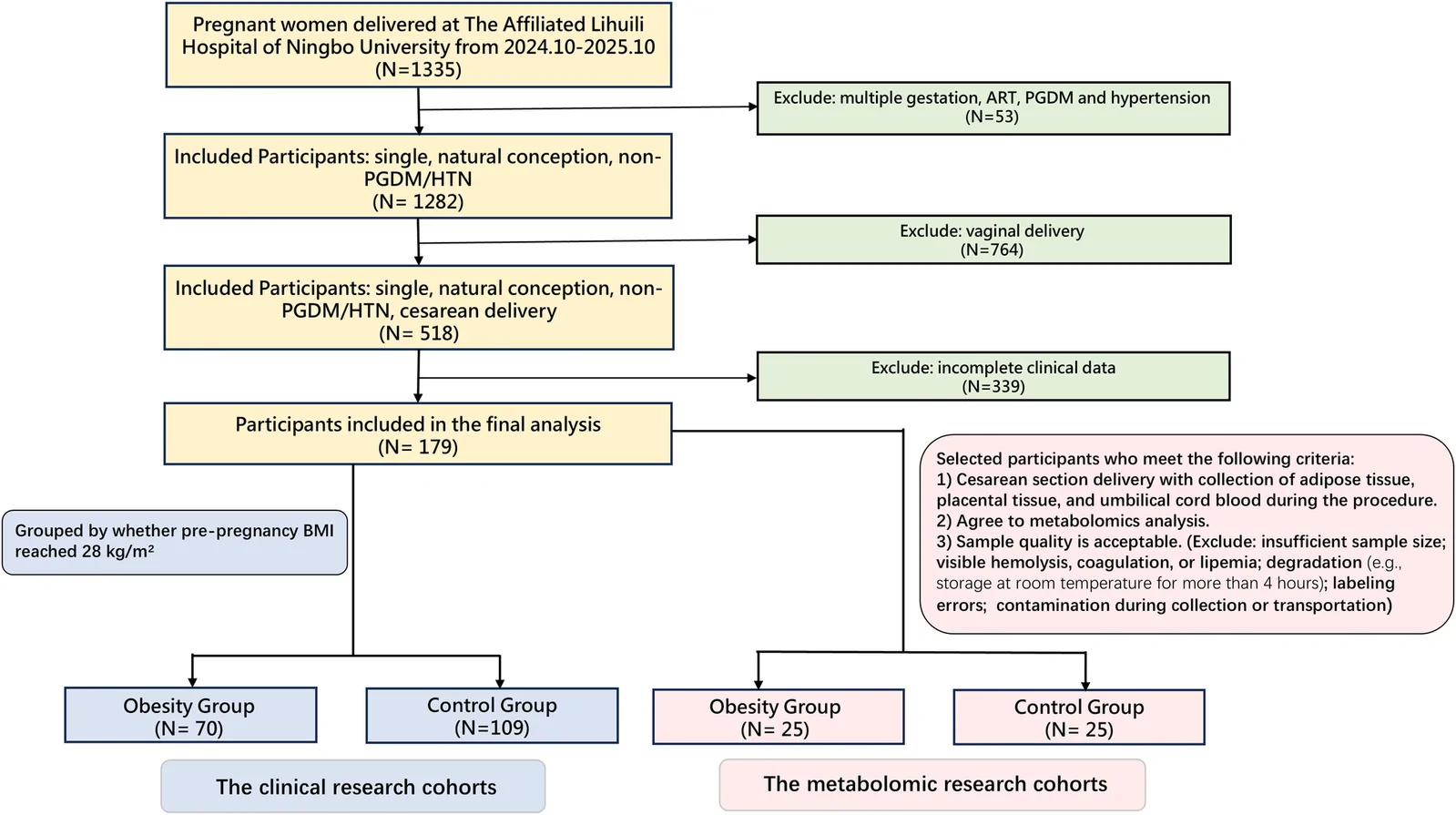
Flow diagram of the study protocol.

This study protocol was approved by the Medical Ethics Committee of The Affiliated Lihuili Hospital of Ningbo University (Approval No. KY2023PJ183), and all participants have provided informed consent.

### 2.2 Clinical data collection and outcomes

General maternal information was collected via the electronic medical record system, including age, gravidity, parity, height, pre-pregnancy weight, census register, and education background. These women were followed fasting blood-glucose (FBG), total bile acids (TBA), blood lipids (total cholesterol, triglyceride, high- and low-density lipoprotein cholesterol), and nutritional status indicators (ferritin, folate, vitamin B12, and 25-hydroxyvitamin D) in first and third trimesters of pregnancy. These indicators were tested in the hospital's biochemical laboratory according to clinical requirements. We also collected their main outcomes, including GDM, HDP, preterm birth (PB), macrosomia, and postpartum hemorrhage (PPH). APOs were defined as the occurrence of any of the above outcomes.

### 2.3 Sample collection

Umbilical cord blood (10 mL) was collected aseptically from the umbilical vein after delivery, clotted for 30–60 min at ambient temperature, and centrifuged at 4 °C (3,000 rpm, 10–15 min) to obtain serum. Peritoneal adipose tissue (1–2 g) was harvested from the preperitoneal area below the umbilicus. Placental tissue (1–2 cm^3^) was obtained from the maternal surface 2–3 cm from the cord insertion site, avoiding infarcts, calcifications, and hemorrhage. Solid specimens were rinsed with pre-chilled saline, blotted dry, and all samples were snap-frozen in liquid nitrogen and stored at −80 °C.

### 2.4 Metabolite extraction

For liquid samples (100 µL serum), extraction used 800 µL acetonitrile:methanol (1:1) with internal standards, vortexed, sonicated (5 °C, 40 kHz, 30 min), precipitated at −20 °C for 30 min, centrifuged (4 °C, 13,000 g, 15 min), dried under nitrogen, re-dissolved in 100 µL acetonitrile:water (1:1), and centrifuged again. For solid samples (100 mg tissue), extraction used 800 µL methanol:water (4:1) with internal standards, ground (−10 °C, 50 Hz, 6 min), sonicated (5 °C, 40 kHz, 30 min), precipitated at −20 °C for 30 min, and centrifuged (4 °C, 13,000 g, 15 min). Supernatants were used for LC-MS/MS.

### 2.5 LC-MS/MS analysis

Analysis used a UHPLC-Orbitrap Exploris 480 system with an ACQUITY HSS T3 column (100 × 2.1 mm, 1.8 µm) at 40 °C. Mobile phases: 0.1% formic acid in water: acetonitrile (2:98) and 0.1% formic acid in acetonitrile, flow rate 0.40 mL/min. ESI source operated in positive and negative modes. Data-dependent acquisition (DDA) covered a mass range of 70–1,050 m/z.

### 2.6 Statistical analysis

Clinical data were analyzed using SPSS v.22.0. Normally distributed data were expressed as mean ± SD and compared by *t*-test; non-normally distributed data as median (Q₁, Q₃) and compared by chi-square test. *P* < 0.05 was considered significant.

Raw LC-MS/MS data were processed with Progenesis QI (Waters Corporation, Milford, USA) software. Metabolic features detected in at least 80% of the samples within any group were retained. After filtering, the minimum value in the data matrix was selected to fill the missing value and each metabolic signature was normalized to the sum. To minimize systematic errors derived from sample preparation and instrument instability, the peak response intensities for each of the three sample types (adipose tissue, placental tissue, and umbilical cord serum samples) were severally normalized using the sum normalization method. The variables of QC samples with relative standard deviation (RSD) > 30% were deleted and performed a log10 logarithmic processing to obtain a final data matrix. Metabolism public database HMDB (http://www.hmdb.ca/) and Metlin (https://metlin.scripps.edu/) database were selected to identify mass spectra of the metabolic features.

The data statistical analysis was performed on Majorbio Cloud Platform (https://cloud.majorbio.com). Firstly, Partial Least Squares Discriminant Analysis (PLS-DA) and 7-fold cross-validation were performed to evaluate model stability. All data were Pareto-scaled (Par) to eliminate dimensional differences among metabolites. And overfitting was assessed via a permutation test with 200 iterations, and a Q^2^ intercept value < 0.00 was considered to indicate that the model was not overfitted. Subsequently, Orthogonal Partial Least Squares Discriminant Analysis (OPLS-DA) and Student's *t*-test was conducted to determine metabolic changes between the obesity group and the control group. OPLS-DA can maximize group separation, and extracted Variable Importance in Projection (VIP) values to rank the contribution of each metabolite to the discrimination. Differential metabolites were screened based on VIP > 1 and *p*-value of Student's *t*-test < 0.05. To control the false discovery rate (FDR), Benjamini-Hochberg procedure was then applied. Metabolites simultaneously satisfying VIP > 1 and FDR < 0.05 were considered statistically robust and selected for further biological interpretation. All the above analyses were carried out using the R package ropls (Version 1.6.2), consistent with the criteria used in previous metabolomic studies (13, 14). Finally, Metabolic enrichment and pathway analysis was based on the Kyoto Encyclopedia of Genes and Genomes (KEGG) database (http://www.genome.jp/kegg/), and statistically significantly enriched pathway was identified using Fisher's exact test through Python packages scipy.stats (https://docs.scipy.org/doc/scipy).

### 2.7 Quantitative real-time PCR (qRT-PCR)

To experimentally validate the KEGG pathway analysis, we performed qRT-PCR on the independent validation cohorts’ placental tissues. The cohort included an obese group(*n* = 10) and a control group(*n* = 10). The construction criteria of this cohort were exactly consistent with those of the original metabolomic research cohorts. RNA extraction was performed using TAKARA 9767 (TaKaRa MiniBEST Universal RNA Extraction Kit), with a gDNA removal step included. The RNA quality was assessed by spectrophotometry (OD260/280 between 1.9–2.2). Reverse transcription was carried out using TOYOBO-FSQ-201 (ReverTra Ace qPCR RT Master Mix). The qPCR reactions were carried out on a LightCycler 480 (Roche) using LightCycler 480 SYBR Green I master SYBR. Reaction mix contained 6 µL SYBR Green premix, 1 µL primer pair (concentration 10 µM), and 3 µL cDNA (diluted 5-fold after extraction). The following primers were used: (1) CYP11A1: Forward 5′-AGGACCAAGACCTGGAAGGA-3′, Reverse 5′-GTCTGTAGAGGATGCCACGG-3′; (2) CYP19A1: Forward 5′-TGCGAGTCTGGATCTCTGGA-3′, Reverse 5′-CCCAAGTTTGCTGCCGAATC-3′; (3) *β*-actin (internal control): Forward 5′-AGACCTGTACGCCAACACAG-3′, Reverse 5′-GTACTTGCGCTCAGGAGGAG-3′. The cycling conditions were: 95 °C for 30 s, followed by 40 cycles of 95 °C for 15 s and 62 °C for 30 s. The specificity of the amplification was verified by melt curve analysis. Relative mRNA expression was calculated using the 2^(−*ΔΔ*Ct) method, and statistical significance was assessed by Student's *t*-test, with *P* < 0.05 considered significant.

## 3 Results

### 3.1 Basic information and laboratory indicators of the pregnant women

This study found no statistically significant differences in age, gravidity, and the incidence of preterm birth between the two groups. However, statistically significant differences were observed in parity, census register and educational background. In the obese cohort, GDM, HDP, and PPH were significantly higher than the control group. Notably, despite no significant difference in the incidence of macrosomia between the groups, there was a statistically significant difference in neonatal weight, as shown in Table 1.

**Table 1:** Basic information of the pregnant women.

Variables	Total (*n* = 179)	Control group (*n* = 109)	Obesity group (*n* = 70)	Statistic	*P*
Age	30.34 ± 4.46	30.06 ± 4.52	30.76 ± 4.37	*t* = −1.01	0.312
Pre-pregnancy BMI(kg/m^2^)	25.24 (20.32, 29.52)	21.10 (19.05, 24.46)	30.79 (28.95, 33.21)	*Z* = −11.28	**<**.001*
Neonatal weight(g)	3,180.28 ± 461.57	3,114.22 ± 378.09	3,283.14 ± 555.17	*t* = −2.23	**0**.027*
Postpartum blood loss(mL)	200 (200, 300)	200 (150, 300)	250 (200, 337)	*Z* = −3.62	**<**.001*
Gravidity, *n*(%)				-	0.527
1	76 (42.46)	51 (46.79)	25 (35.71)		
2	48 (26.82)	30 (27.52)	18 (25.71)		
3	30 (16.76)	15 (13.76)	15 (21.43)		
4	14 (7.82)	8 (7.34)	6 (8.57)		
5	6 (3.35)	3 (2.75)	3 (4.29)		
6	5 (2.79)	2 (1.83)	3 (4.29)		
Parity, *n*(%)				-	**0**.046*
0	119 (66.48)	80 (73.39)	39 (55.71)		
1	47 (26.26)	22 (20.18)	25 (35.71)		
2	12 (6.70)	6 (5.50)	6 (8.57)		
3	1 (0.56)	1 (0.92)	0 (0.00)		
Census register, *n*(%)				*χ*^2^ = 8.85	**0**.003*
1	73 (40.78)	54 (49.54)	19 (27.14)		
2	106 (59.22)	55 (50.46)	51 (72.86)		
Education background, *n*(%)				*χ*^2^ = 16.69	**<**.001*
1	119 (66.48)	83 (76.15)	36 (51.43)		
2	25 (13.97)	15 (13.76)	10 (14.29)		
3	35 (19.55)	11 (10.09)	24 (34.29)		
Preterm birth, *n*(%)				*χ*^2^ = 0.88	0.349
1	16 (8.94)	8 (7.34)	8 (11.43)		
2	163 (91.06)	101 (92.66)	62 (88.57)		
GDM, *n*(%)				*χ*^2^ = 16.08	**<**.001*
1	43 (24.02)	15 (13.76)	28 (40.00)		
2	136 (75.98)	94 (86.24)	42 (60.00)		
HDP, *n*(%)				*χ*^2^ = 17.96	**<**.001*
1	28 (15.64)	7 (6.42)	21 (30.00)		
2	151 (84.36)	102 (93.58)	49 (70.00)		
Fetal macrosomia, *n*(%)				*χ*^2^ = 3.09	0.079
1	8 (4.47)	2 (1.83)	6 (8.57)		
2	171 (95.53)	107 (98.17)	64 (91.43)		

*t*, *t*-test; *Z*, Mann–Whitney test; *χ*^2^, Chi-square test; -, Fisher exact.

Census register: 1 represents the population within Zhejiang Province, and 2 represents the population outside Zhejiang Province.

Education background: 1 indicates university degree or above, 2 indicates high school degree, 3 indicates junior high school or primary school degree.

Premature birth, GDM, HBP, Fetal macrosomia: 1 indicates being ill, 2 indicates no.

*P ＜ 0.05.

FBG, TBA, blood lipids, vitamin B12 and 25-hydroxyvitamin D during early pregnancy, FBG, total cholesterol (TC) and high-density lipoprotein cholesterol (HDL-C) during late pregnancy were significantly higher in the obesity group than in the control group. All other measured parameters showed no statistically significant differences, as detailed in Table 2.

**Table 2:** Laboratory indicators of the pregnant women.

Variables	Total (*n* = 179)	Control group (*n* = 109)	Obesity group (*n* = 70)	Statistic	*P*
First trimester of pregnancy					
TC (nmol/L)	4.56 ± 0.77	4.47 ± 0.75	4.71 ± 0.78	*t* = −2.04	**0**.043*
FBG (nmol/L)	4.78 (4.56, 4.99)	4.72 (4.55, 4.88)	4.91 (4.62, 5.07)	*Z* = −3.09	**0**.002*
TBA (*μ*mol/L)	1.50 (1.10, 2.10)	1.40 (1.08, 2.00)	1.90 (1.20, 2.18)	*Z* = −2.05	**0**.041*
TG (nmol/L)	1.38 (1.14, 1.70)	1.30 (1.10, 1.62)	1.53 (1.25, 1.84)	*Z* = −3.01	**0**.003*
HDL-C (nmol/L)	1.51 (1.25, 1.64)	1.57 (1.36, 1.80)	1.25 (1.12, 1.54)	*Z* = −5.21	**<**.001*
LDL-C (nmol/L)	2.36 (1.98, 2.71)	2.20 (1.89, 2.57)	2.65 (2.31, 2.99)	*Z* = −4.96	**<**.001*
Ferritin (μg/L)	30.80 (19.05, 70.53)	30.80 (18.40, 62.90)	32.00 (21.13, 84.21)	*Z* = −1.16	0.244
Folate (nmol/L)	26.05 (23.95, 33.01)	26.05 (21.76, 33.01)	26.05 (26.05, 26.05)	*Z* = −1.35	0.177
Vitamin B12 (nmol/L)	235.00 (187.00, 246.00)	235.00 (235.00, 265.00)	187.00 (187.00, 218.00)	*Z* = −8.06	**<**.001*
25-hydroxyvitamin D (ng/mL)	21.37 (13.88, 25.20)	21.39 (15.90, 26.45)	19.08 (13.84, 24.04)	*Z* = −2.02	**0**.043*
Third trimester of pregnancy					
TC (nmol/L)	6.11 ± 1.07	6.27 ± 1.07	5.87 ± 1.04	*t* = 2.46	**0**.015*
FBG (nmol/L)	4.64 (4.25, 5.67)	4.56 (4.20, 5.35)	4.75 (4.35, 5.87)	*Z* = −1.98	**0**.048*
TBA (μmol/L)	2.70 (1.90, 4.20)	2.90 (2.00, 4.30)	2.60 (1.72, 3.95)	*Z* = −1.09	0.276
TG (nmol/L)	3.69 (2.83, 4.86)	3.52 (2.69, 4.78)	3.91 (3.08, 4.97)	*Z* = −1.62	0.105
HDL-C (nmol/L)	1.90 (1.65, 2.16)	2.00 (1.72, 2.18)	1.79 (1.57, 2.08)	*Z* = −2.66	**0**.008*
LDL-C (nmol/L)	3.23 (2.67, 3.72)	3.25 (2.74, 3.87)	3.15 (2.54, 3.63)	*Z* = −1.21	0.227
Ferritin (μg/L)	17.05 (10.30, 27.15)	17.30 (11.30, 28.80)	16.85 (9.25, 24.35)	*Z* = −1.07	0.283
Folate (nmol/L)	21.62 (13.39, 34.55)	21.62 (13.70, 36.85)	19.17 (13.07, 30.04)	*Z* = −1.46	0.143
Vitamin B12 (nmol/L)	205.00 (176.00, 254.00)	205.00 (179.00, 274.00)	205.00 (170.50, 231.25)	*Z* = −1.58	0.113
25-hydroxyvitamin D (ng/mL)	24.74 (21.89, 27.93)	24.74 (22.02, 27.93)	24.70 (20.71, 27.82)	*Z* = −0.52	0.602

*t*, *t*-test; *Z*, Mann–Whitney test.

*P ＜ 0.05.

### 3.2 Adipose tissue metabolomics results

PLS-DA results (Figure 2A) showed a separate trend between groups, and QC samples were tightly clustered, indicating good reproducibility of the experiment. Additionally, the permutation test (*R*^2^ intercept = 0.5314, *Q*^2^ intercept = −0.4943; Figure 2B) suggested that the model was stable and showed no overfitting. OPLS-DA results (Figure 2C) revealed a clear separation, indicating significant differences between the two groups. Using the threshold of VIP > 1 and Student's *t*-test p < 0.05, 319 differential metabolites were identified (219 up-regulated and 100 down-regulated in the obesity group); after FDR correction (FDR < 0.05), 87 metabolites remained significant (63 up-regulated and 24 down-regulated; Supplementary Table S1). Random Forest algorithms were employed to assess the discriminatory power of metabolites on pregnancy outcomes. Model validation using Recursive Feature Elimination with Cross-Validation (RFECV; Figure 2D) showed decreasing error rates with increasing features, stabilizing at the top 8 features. Variable importance ranking (Figure 2E) identified Vitamin B12, etiocholanolone, folate, L-alanine, TC, betaine, and LDL-C as top contributors. Compared with the control group, KEGG enrichment analysis (Figure 2F) highlighted pathways central to energy and amino acid metabolism in the obesity group, including arginine and proline metabolism, the pentose phosphate pathway, and cysteine and methionine metabolism. Additionally, several signaling pathways related to endocrine and neuronal regulation (e.g., vitamin B6 metabolism, cGMP-PKG, cAMP, and GABAergic synapse) were found to be enriched. Collectively, these enriched pathways reflect characteristics of disordered energy metabolism and dysregulated endocrine signaling in maternal peripheral metabolism. Correlation analysis (Figure 2G) revealed positive associations of lipid and steroid metabolites (etiocholanolone, TC, LDL-C) with clinical indicators, whereas betaine and L-alanine showed negative correlations. These findings indicate a direct association between maternal peripheral metabolic dysregulation and clinical phenotypes.

**Figure 2:**
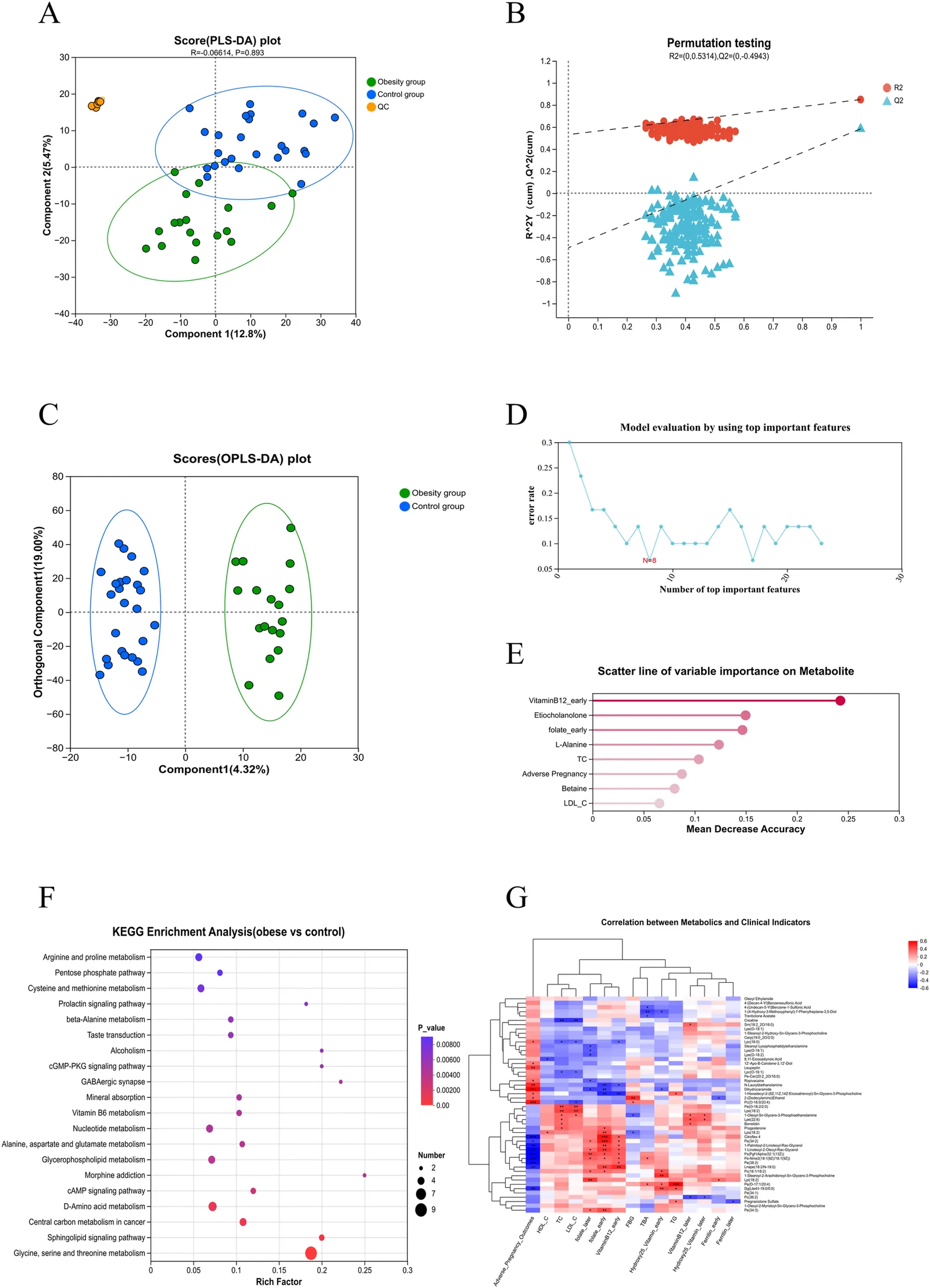
Adipose tissue metabolomics results. **(A)** PLS-DA score plot showing the separation among the obesity, control, and QC groups. **(B)** PLS-DA permutation testing (200 random permutations); dashed lines represent regression lines for *R*^2^ and *Q*^2^, and the *Q*^2^ intercept < 0.00 confirmed the model without overfitting. **(C)** OPLS-DA score plot for group discrimination. **(D)** Recursive Feature Elimination with Cross-Validation plot showing the cross-validation error rate of the random forest model as a function of the number of selected metabolites; the point corresponding to the lowest error rate indicates the optimal feature subset. **(E)** Random Forest variable importance result plot showing the top 8 metabolites ranked by variable importance; larger *X*-axis values indicate greater contribution of the metabolite to the model's classification performance, and darker colors correspond to higher importance. **(F)** KEGG pathway enrichment bubble plot of the identified differential metabolites. **(G)** Correlation heatmap showing the association network between key differential metabolites and clinical indicators; *0.01 < *P* ≤ 0.05, **0.001 < *P* ≤ 0.01, ***P ≤ 0.001.

### 3.3 Placental tissue metabolomics results

Similar analyses were performed on placental tissue. PLS-DA results (Figure 3A) revealed a clustering trend in QC samples and clear separation between groups indicating good model stability. The permutation test (*R*^2^ intercept = 0.8718, *Q*^2^ intercept = −0.0972; Figure 3B) showed no overfitting. OPLS-DA results (Figure 3C) demonstrated distinct group separation. A total of 331 differential metabolites were identified using thresholds of VIP > 1 and Student's *t*-test *p* < 0.05 (218 up-regulated, 113 down-regulated); after FDR correction (FDR < 0.05), 99 remained significant (70 up-regulated, 29 down-regulated; Supplementary Table S2). Random forest validation (Figure 3D) indicated that the top 8 features achieved low error rates, stabilizing at 16 features. Variable importance ranking (Figure 3E) revealed that the top-ranking metabolites, in order, were Vitamin B12, folate, L-proline, 16-glucuronide-estriol, 25-hydroxyvitamin D, TC, and choline sulfate. KEGG enrichment analysis (Figure 3F) identified the steroid hormone biosynthesis, ovarian steroidogenesis and folate biosynthesis as significantly perturbed nodes. Additional enriched pathways encompassed various core metabolic processes (e.g., pentose phosphate pathway, beta-alanine metabolism, linoleic acid metabolism) as well as diverse signaling and physiological regulation pathways (e.g., oxytocin signaling, regulation of lipolysis in adipocytes and aldosterone synthesis and secretion). This enrichment pattern underscores the placenta's role as a hormone-synthesizing organ and suggests that placental steroid metabolism and one-carbon metabolism abnormalities serve as critical links between maternal metabolic status and fetal outcomes. Correlation analysis (Figure 3G) revealed extensive networks linking steroid metabolites (16-glucuronide-estriol, progesterone) and lipid metabolites (stearoyl lysophosphatidylethanolamine) with clinical metrics, highlighting functional coupling between placental lipid metabolism and steroidogenesis.

**Figure 3:**
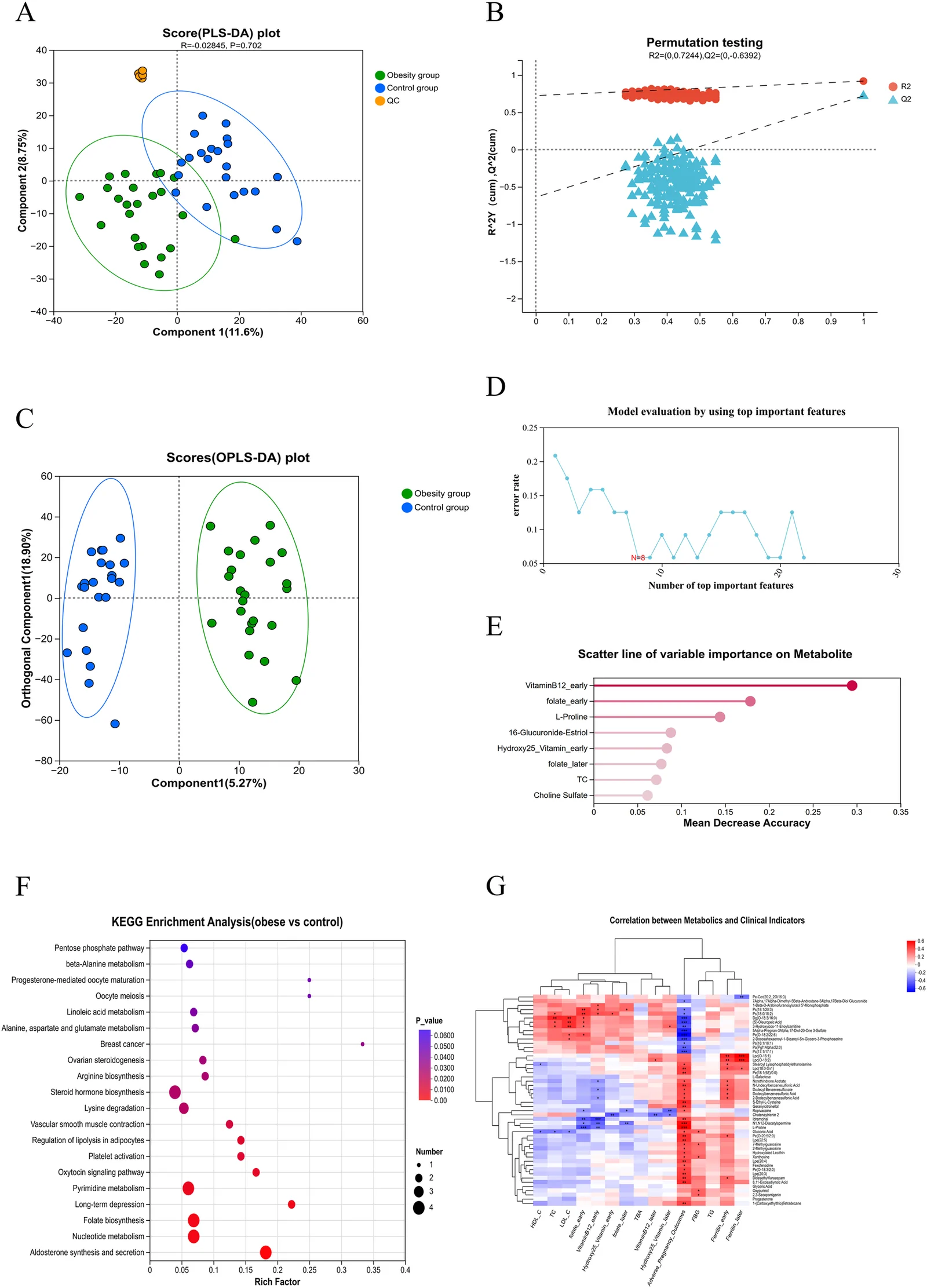
Placental tissue metabolomics results. **(A)** PLS-DA score plot showing the separation among the obesity, control, and QC groups. **(B)** PLS-DA permutation testing (200 random permutations); dashed lines represent regression lines for *R*^2^ and *Q*^2^, and the *Q*^2^ intercept < 0.00 confirmed the model without overfitting. **(C)** OPLS-DA score plot for group discrimination. **(D)** Recursive Feature Elimination with Cross-Validation plot showing the cross-validation error rate of the random forest model as a function of the number of selected metabolites; the point corresponding to the lowest error rate indicates the optimal feature subset. **(E)** Random Forest variable importance result plot showing the top 8 metabolites ranked by variable importance; larger *X*-axis values indicate greater contribution of the metabolite to the model's classification performance, and darker colors correspond to higher importance. **(F)** KEGG pathway enrichment bubble plot of the identified differential metabolites. **(G)** Correlation heatmap showing the association network between key differential metabolites and clinical indicators; *0.01 < *P* ≤ 0.05, **0.001 < *P* ≤ 0.01, ***P ≤ 0.001.

### 3.4 Umbilical cord blood serum metabolomics results

Extending the analytical framework to fetal circulation, we next examined umbilical cord blood serum. PLS-DA and the permutation test (*R*^2^ intercept = 0.8704, *Q*^2^ intercept = −0.4031; Figures 4A,B) showed the model was stable and the results were reproducible. OPLS-DA results (Figure 4C) showed clear separation between groups. Using the thresholds of VIP > 1 and Student's *t*-test *p* < 0.05, 185 differential metabolites were identified (105 up-regulated, 80 down-regulated); after FDR correction (FDR < 0.05), 15 remained significant (4 up-regulated, 11 down-regulated; Supplementary Table S3). Random forest validation (Figure 4D) demonstrated the top 3 features achieved low error rates, stabilizing at approximately 10 features. Variable importance ranking (Figure 4E) showed vitamin B12, folate, and 5*α*-androsterone as the top three discriminatory metabolites. The highest enrichment levels were exhibited by steroid hormone biosynthesis, the TCA cycle, and the glutamatergic synapse in KEGG enrichment analysis (Figure 4F). Additional enriched pathways encompassed a wide range of metabolic and signaling processes (e.g., amino acid and vitamin metabolism, GnRH and serotonergic signaling, ferroptosis, and ABC transporters). These findings indicate that alterations in the cord blood metabolic profile reflect dysregulation of energy metabolism, neurotransmitter metabolism, and steroid hormone metabolism in fetal circulation under maternal obesity. Correlation analysis (Figure 4G) revealed that cord blood metabolites comprehensively reflect multidimensional metabolic pathway dysregulation in fetal circulation under maternal obesity and are closely correlated with clinical indicators, providing important evidence for elucidating the metabolic regulatory mechanisms underlying pregnancy outcomes. For example, steroid hormone-related metabolites (5*α*-androsterone, 16-glucuronide-estriol, dehydroepiandrosterone sulfate derivatives) and amino acid and energy metabolites (L-glutamine, L-phenylalanine) showed significant correlations with clinical indicators, reinforcing the central roles of androgen disorders and TCA cycle abnormalities in obesity-related adverse pregnancy outcomes.

**Figure 4:**
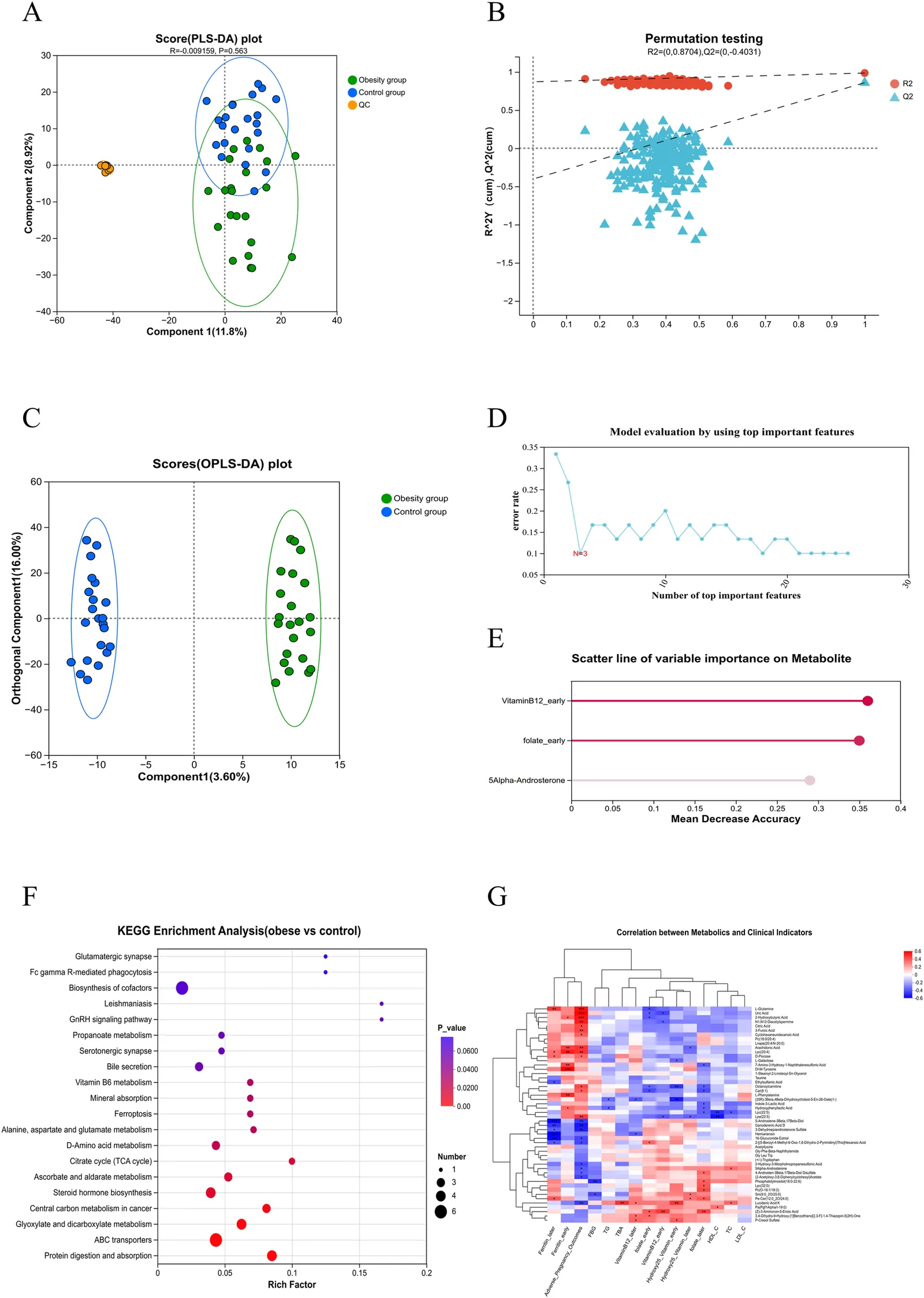
Umbilical cord blood serum metabolomics results. **(A)** PLS-DA score plot showing the separation among the obesity, control, and QC groups. **(B)** PLS-DA permutation testing (200 random permutations); dashed lines represent regression lines for *R*^2^ and *Q*^2^, and the *Q*^2^ intercept < 0.00 confirmed the model without overfitting. **(C)** OPLS-DA score plot for group discrimination. **(D)** Recursive Feature Elimination with Cross-Validation plot showing the cross-validation error rate of the random forest model as a function of the number of selected metabolites; the point corresponding to the lowest error rate indicates the optimal feature subset. **(E)** Random Forest variable importance result plot showing the top 3 metabolites ranked by variable importance; larger *X*-axis values indicate greater contribution of the metabolite to the model's classification performance, and darker colors correspond to higher importance. **(F)** KEGG pathway enrichment bubble plot of the identified differential metabolites. **(G)** Correlation heatmap showing the association network between key differential metabolites and clinical indicators; *0.01 < *P* ≤ 0.05, **0.001 < *P* ≤ 0.01, ***P ≤ 0.001.

### 3.5 qRT-PCR results

Given that the placenta serves as a critical link between maternal metabolic status and fetal outcomes and possesses hormone synthesis capabilities, playing a pivotal role in the maternal-fetal axis, we selected placenta tissue as the subject for our validation experiments. To experimentally corroborate the KEGG-enriched steroid hormone biosynthesis pathway, we performed qRT-PCR to examine the mRNA expression of two rate-limiting enzymes in this pathway, cholesterol side-chain cleavage enzyme (CYP11A1) and aromatase (CYP19A1), in the independent external cohort of placental tissues (*n* = 10 per group). *β*-actin was used as the internal reference gene, and the specificity of each primer pair was verified by melt curve analysis (Supplementary Table S4).

As shown in Figure 5, the relative mRNA expression of both CYP11A1 and CYP19A1 was significantly down-regulated in the obesity group compared with the control group [CYP11A1: 0.62 ± 0.43 vs.1.14 ± 0.62, *P* < 0.05; CYP19A1: 1.09(0.76, 1.24) vs. 0.56(0.31, 0.76), *P* < 0.05; Supplementary Table S4]. Notably, this transcriptional down-regulation was directionally consistent with the decreased abundances of related steroid metabolites in our placental metabolomic data, such as 16-glucuronide-estriol (FDR < 0.05, down-regulated; Supplementary Table S2).

**Figure 5:**
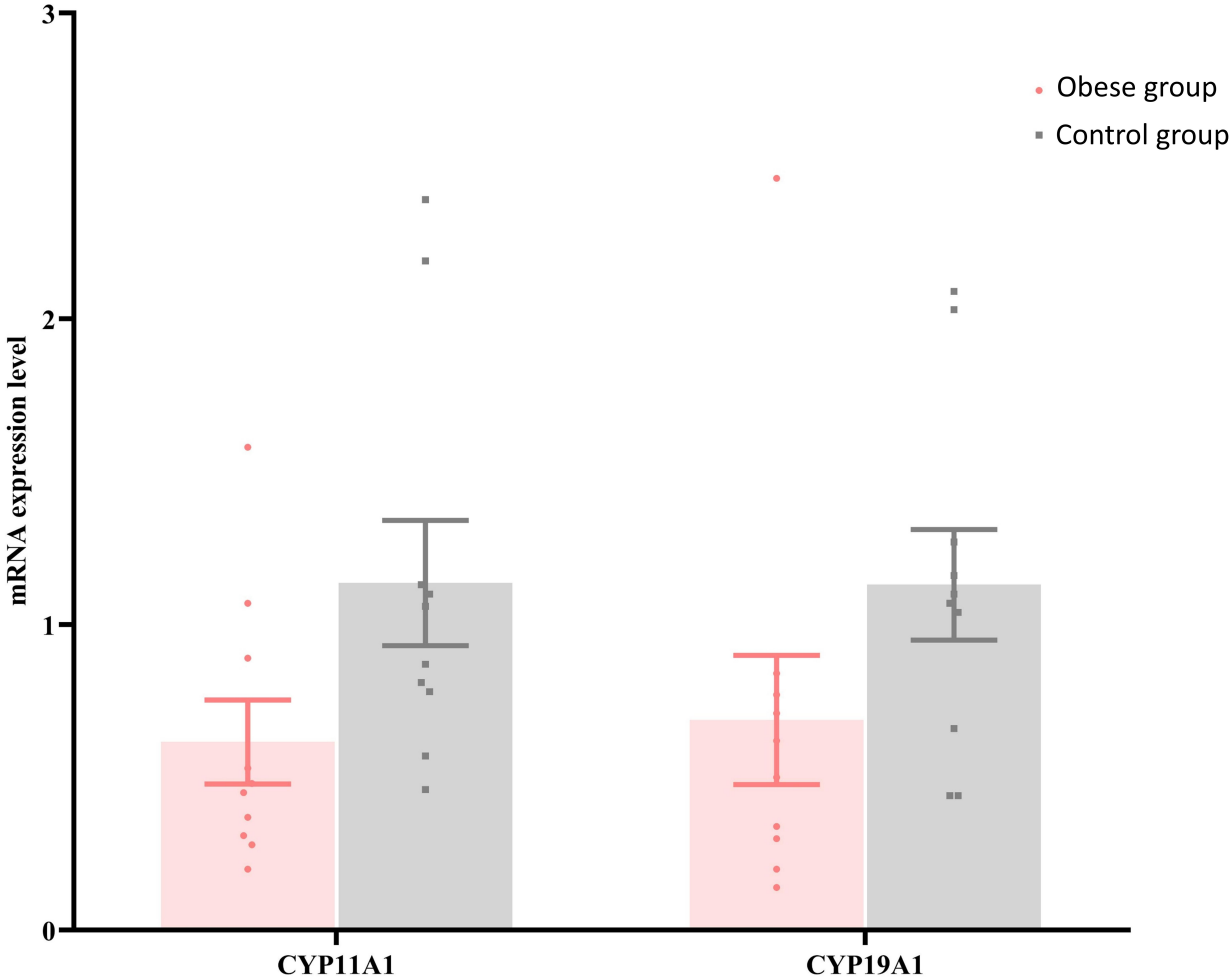
Placental tissue qPT-PCR results. The Placental tissue qPT-PCR results bar chart showing the relative mRNA expression of CYP11A1 and CYP19A1 [CYP11A1: 0.62 ± 0.43 vs.1.14 ± 0.62, *P* = 0.026; CYP19A1: 1.09(0.76, 1.24) vs. 0.56(0.31, 0.76), *P* = 0.045].

## 4 Discussion

This exploratory study integrates metabolomic data from maternal adipose tissue, placenta, and umbilical cord blood to elucidate the hierarchical characteristics and potential mechanistic clues of metabolic dysregulation at the maternal-fetal interface in maternal obesity. Using Random Forest, KEGG enrichment, and metabolite-clinical indicator correlation analysis, we identified three key findings: (1) Vitamin B12 and folic acid emerged as the most critical classification metabolites across all three sample sites, suggesting that one-carbon metabolism abnormalities pervade the entire maternal-fetal axis. (2) Placental tissue showed significant enrichment in the “steroid hormone biosynthesis” pathway, with metabolites such as estriol derivatives and progesterone strongly correlating with clinical outcomes. This indicates that placental steroid metabolism dysfunction serves as a central hub connecting maternal obesity to adverse pregnancy outcomes. (3) In addition to the steroid hormone pathway, fetal circulation in the umbilical cord blood also exhibited significant enrichment in pathways such as the tricarboxylic acid cycle and glutamatergic synapse, reflecting dysregulation in energy metabolism and neurotransmitter metabolism within the fetal circulation.

In our study, vitamin B12 and folate ranked prominently among the variables in adipose tissue, placenta, and umbilical cord blood, aligning with previous research suggesting that “insufficient maternal methyl donors can lead to placental dysfunction and adverse pregnancy outcomes” (15, 16). Vitamin B12 and folate, as critical cofactors in the methionine cycle, are essential for maintaining normal physiological processes. Their deficiency can lead to elevated homocysteine and altered DNA methylation patterns, subsequently impacting placental hormone synthesis and fetal growth (15, 17). Notably, the “folate biosynthesis” pathway was also specifically enriched in placental tissue, while umbilical cord blood retained a high importance of vitamin B12 and folate. This suggests a gradient transfer and dependency of one-carbon metabolites between the mother, placenta, and fetus. Our findings support the notion that folate and vitamin B12 supplementation during pregnancy not only prevents neural tube defects but may also improve pregnancy outcomes by modulating placental steroid metabolism (18). The central role of B12/folate dysfunction in our study is further corroborated by findings from an elderly cohort, in which the same metabolic disturbance was identified as an independent risk factor for sarcopenia (19). This cross-population consistency suggests that one-carbon metabolic disruption represents a common pathophysiological hub across different life stages and disease contexts, reinforcing the importance of vitamin B12 and folate supplementation.

We acknowledge that our KEGG enrichment analysis identified several pathways that appear superficially unrelated to pregnancy outcomes, such as neural and cell signaling related pathways (e.g., cGMP-PKG, cAMP, GABAergic synapse, alcoholism, and morphine addiction). This pattern is commonly observed in untargeted metabolomics and can be attributed to two factors. Firstly, key signaling molecules such as cAMP and cGMP play pleiotropic roles: they are not only associated with metabolic and endocrine dysregulation, but are also components of pathways related to central nervous system functions. Secondly, a single metabolite mapped to multiple KEGG entries due to its involvement in interconnected reactions further contributes to the dispersion of enrichment signals. Collectively, these factors contribute to the algorithmic enrichment of seemingly unrelated pathways, without implying any pathological relevance to specific addictive behaviors or neurological conditions. Therefore, while we report these findings for completeness, our biological interpretation prioritizes the consistently altered and functionally validated pathways across all three compartments. In the core discussion, we prioritize biologically relevant, cross-tissue consistent pathways: one-carbon metabolism dysfunction, placental steroid hormone biosynthesis dysregulation and fetal energy and neurotransmitter metabolism disturbances.

The placenta exhibits tissue-specific enrichment of pathways involved in steroid hormone biosynthesis with metabolites such as 16-glucuronide estriol and progesterone showing significant correlations with clinical indicators. This finding aligns perfectly with the established endocrine functions of the placenta. The placenta synthesizes hormones such as estriol and progesterone to maintain pregnancy (20), whereas chronic inflammation and insulin resistance associated with maternal obesity can interfere with the expression of placental steroidogenic enzymes [e.g., cholesterol side-chain cleavage enzyme(CYP11A1), aromatase(CYP19A1)] (21–23). This study using qPR-PCR found that placental steroidogenic enzymes exhibit reduced expression at the transcriptome level, which is consistent with the downregulation trend of 16-glucuronide estriol in metabolomics. However, the KEGG analysis prompted us to consider a homeostatic perturbation. Notably, we found progesterone was up-regulated (*P* < 0.05; Supplementary Table S2), and one interpretation is that reduced CYP19A1 may impede aromatization, potentially creating a bottleneck that leads to progesterone accumulation without restoring downstream output. While speculative and requiring flux validation, this model offers a coherent reconciliation between KEGG enrichment and the net suppressive direction. Collectively, these findings suggest that pre-pregnancy obesity may impair the biosynthesis of placental steroid hormones, leading to a perturbed metabolic state, and indicating that improving placental steroid metabolism could potentially help reduce adverse pregnancy outcomes in pregnant women with pre-pregnancy obesity. This study further revealed a significant presence of etiocholanolone (an androgen metabolite) in adipose tissue, with 5*α*-androsterone ranking highest in umbilical cord blood. This suggests that androgen metabolites may serve as intermediates or bypass products in the steroid hormone synthesis pathway, potentially transmitting aberrant signals between the maternal-placental-fetal unit. This finding is consistent with literature reports indicating that excessive androgens during pregnancy can lead to placental insufficiency and abnormal fetal growth (24).

Majidi et al. reported elevated short-chain and hydroxylated long-chain acylcarnitines in GDM and PGDM pregnancies, pointing to fatty acid *β*-oxidation disturbances (25). Our study extends these observations by revealing concurrent one-carbon and steroid hormone biosynthesis disruptions across the maternal-placental-fetal axis in pre-pregnancy obesity, suggesting that fatty acid oxidation, methyl transfer, and steroidogenesis are jointly disrupted in metabolically complicated pregnancies. Beyond steroid hormone biosynthesis, cord blood also showed the TCA cycle, glutamatergic and serotonergic synapses, ferroptosis, and ABC transporters enrichment. This indicates that maternal obesity not only influences fetal circulatory hormone levels but also impacts energy metabolism, neurotransmitter metabolism, and oxidative stress. The enrichment of glutamatergic and serotonergic synapses is particularly noteworthy, given the crucial roles these neurotransmitters play in fetal brain development (26, 27). This suggests that maternal obesity may exert long-term effects on offspring neurodevelopment through alterations in the umbilical cord blood metabolomic profile. Significant associations observed in correlation analyses between amino acid metabolites (e.g., L-glutamine and L-phenylalanine) and clinical indicators lend additional support to these findings. Collectively, these results indicate that cord blood metabolites can comprehensively reflect multi-dimensional metabolic pathway dysregulation in fetal circulation under maternal obesity, providing important evidence for elucidating the metabolic regulatory mechanisms underlying pregnancy outcomes. Our pathway-centric analytical framework aligns with that of Dehghanbanadaki et al., who identified amino acid metabolic pathways as key signatures in osteoporosis using comparable methods (13).

Integrating results, we construct a hierarchical metabolic regulatory network from mother to fetus. Maternal adipose tissue supplies abnormal metabolic substrates (e.g., cholesterol, alanine) and hormonal precursors (e.g., etiocholanolone). The placenta converts these signals into active hormones (e.g., estriol and progesterone) via steroid hormone biosynthesis pathways. Umbilical cord blood then reflects the fetal ultimate response, manifesting as extensive disorders in energy metabolism, neurotransmitter metabolism, and oxidative stress (28). Vitamin B12 and folate levels and the activity of steroid hormone synthesis enzymes, as the key nodes within this network, may serve as future targets for intervention. In contrast to previous studies that solely analyzed placental or umbilical cord blood, the multi-site integrated design of our study depicts the complete maternal-fetal metabolic axis map for the first time.

This study has several limitations. First, the sample size is relatively limited, and the stability of the random forest model requires validation in an independent external cohort. Second, the metabolomic data are from a cross-sectional design, precluding causal inference; the temporal relationship between maternal obesity and metabolite alterations needs clarification through animal studies or prospective cohorts. Third, clinical indicators primarily focused on adverse pregnancy outcomes and did not detail specific subtypes such as preeclampsia, GDM, or fetal growth restriction, thereby limiting subgroup analyses. Fourth, we have validated the mRNA expression of two key steroidogenic enzymes, CYP11A1 and CYP19A1, in placental tissues by qRT-PCR. Future studies may extend this validation to protein-level and multi-tissue analyses to further elucidate the mechanistic landscape. Fifth, sociodemographic differences (parity, census register, education) observed in the full clinical cohort could not be adjusted for in the metabolomic research cohort, where the limited sample size (25 per group) resulted in insufficient cases per stratum for reliable stratification or regression analysis (Supplementary Table S5). Future large-scale prospective cohorts with adequate statistical power are warranted to disentangle the independent effects of maternal obesity from these sociodemographic confounders and to establish robust predictive models.

In conclusion, this study integrates metabolomic data from maternal adipose tissue, placenta, and umbilical cord blood and maps the maternal-fetal metabolic axis in adverse pregnancy outcomes associated with maternal obesity. Aberrant one-carbon metabolism, driven by deficiencies in vitamin B12 and folate, represents a common upstream event across all three compartments. Dysregulation of placental steroid hormone biosynthesis serves as a crucial nexus linking maternal metabolic disturbances to fetal outcomes. Furthermore, perturbations in energy metabolism, neurotransmitter metabolism, and oxidative stress within the umbilical cord blood reflect the ultimate fetal circulatory response. Three compartment-specific metabolites (etiocholanolone in adipose tissue, 16-glucuronide-estriol in placenta, and 5*α*-androsterone in cord blood) were identified as potential biomarkers. Clinically, monitoring vitamin B12, folate, and steroid-related metabolites during pregnancy may enable early identification of high-risk individuals, and targeting placental steroid metabolism could offer a therapeutic strategy to reduce adverse pregnancy outcomes and improve offspring neurodevelopment.

## Data Availability

The datasets presented in this study can be found in online repositories. The names of the repository/repositories and accession number(s) can be found in the article/Supplementary Material. The data presented in the study are deposited in the OMIX, China National Center for Bioinformation https://ngdc.cncb.ac.cn, accession number: OMIX019141.
